# Determinants of farmers’ adaptation strategies to climate change impacts in northwestern Ethiopia

**DOI:** 10.1016/j.heliyon.2023.e18514

**Published:** 2023-07-20

**Authors:** Esubalew Molla, Yoseph Melka, Getnet Desta

**Affiliations:** a Department of Environmental Science, College of Natural Resource and Environmental Science, Oda Bultum University, Chiro, Ethiopia; b Department of Natural Resource Economics and Policy, College of Forestry and Natural Resources, Hawassa University, Hawassa, Ethiopia

**Keywords:** Adaptation, Agro-ecologies, Climate change, Multinomial logit regression, Bure Zuria district

## Abstract

Climate change and related extreme events have become global challenges in ensuring sustainable development. This affects water availability and agricultural production, particularly in developing countries. This study explored the factors affecting farmers' adaptation mechanisms to climate change in different agro-ecological zones of the Bure Zuria district of northwestern Ethiopia. A household survey, focus group discussion, key informant interviews, and observations were used to acquire primary data on farmers' socioeconomic and demographic characteristics, adaptation strategies and potential barriers. About 190 randomly selected households from different agro-ecologies were included in this study. Moreover, reports and published sources were used to acquire secondary data. Data were analyzed using a multinomial logit regression model and descriptive statistics. The results indicated that soil and water conservation practices (26.7%) were the main adaptation responses in highland agro-ecology. In contrast, supplementary feeding for livestock (56%) was the main adaptation response to the adverse effects of climate extremes in lowland agro-ecology. Farmers identified land scarcity (25.84%) and shortage of water for irrigation (28.57%) as major barriers to adaptation in the highland and lowland agro-ecologies, respectively. In addition, agro-ecology, education level, age, active labor, number of livestock (TLU), off-farm income, frequency of extension contacts, credit access, and market access were decisive factors affecting farmers’ adaptation mechanisms to withstand extreme climatic events. In conclusion, soil and water conservation practices are more practiced in highland areas than in lowland areas. Because the steepness of farmlands and erodible soils increase farmers' vulnerability to flood hazards in highland areas than in lowland areas. It is suggested that investigations on the climate change-induced gender-differentiated impacts shall be conducted to design all-inclusive and effective responses.

## 1 Introduction

Though climate change and related hazards are experienced all over the world, it is significant in socioeconomic groups whose adaptive capacity is low, especially the poor and marginalized societies [[1], [2], [3]]. Several studies [4,5] show that rural people of poor nations are highly susceptible since their economic activities are largely dependent on climate-sensitive natural resources. The projections of variations in rainfall and temperature indicate intensified extreme weather, which would threaten the global progress toward poverty alleviation by affecting smallholder agriculture [6,7]. Hence, effective early warning systems are vital to lessen the impacts of projected climate change on the most vulnerable socioeconomic groups.

The agriculture sector is an essential driver of economic growth in Africa. Nevertheless, it is predicted to be adversely impacted by climate variability/change due to the continent's dependence on rain-fed agriculture and low adaptive capacity [8,9]. Like most sub-Saharan Africa (SSA) countries, Ethiopia is among the highly vulnerable countries to climate change [10]. Specifically, the farmers' livelihood in the Bure Zuria district is facing great pressure due to climate-related extremes. Bure Zuria district experienced temperature increments and irregular rainfall patterns. Especially, the rain pattern has become more irregular in recent times in the study area. Due to this, farmers are exposed to water shortages throughout the year. This in turn results in a high incidence of human and livestock water-borne diseases. Especially, the frequent incidence of disease in pepper causes significant yield losses [11]. Thus, climate-induced hazards have imposed substantial threat on the agriculture sector and livelihood of the people in the district.

The inability of farmers to adapt to climate change threats aggravates global food insecurity. The future sustainability of the agriculture sector relies on the kinds of adaptation implemented [12]. As per the reports of [13], adaptation to climate change is a practice of adjustment to the actual or projected climate and its impacts, to reduce harm or exploit valuable opportunities. It can lessen the negative influences of climate change [14]. Adaptation measures can be implemented at the individual level, national level, and international levels [15]. In developing countries, implementing effective adaptation mechanisms in agriculture is vital for improving the livelihoods of the poor [16]. The most commonly used adaptation mechanisms are crop diversification, using improved crop varieties, soil conservation, adjusting sowing or planting time, and irrigation [17].

Studies revealed that farmers' adaptation choices can be influenced by various socio-economic and biophysical factors [18,19]. Some of these include sex, age, education, agro-ecology, livestock holding, off-farm income, access to credit, and extension services. The majority of studies in Ethiopia used binary regression models for analysis [12,20,21]. This situation could not permit analyzing decisions greater than two categories. According to Ref. [22], farmers living in diverse agro-ecologies are likely to implement different adaptation options against the pressures of climate change. Nevertheless, the information on the determinants of farmers' choice of adaptation strategies in different agro-ecologies is limited in the study area. Site-specific investigations are vital since farmers' adaptation measures vary spatially. Hence, this research investigated farmers’ adaptation measures and determinants of adaptation mechanisms to climate change hazards in different agro-ecology of the Bure Zuria district.

## 2 Methodology

### 2.1 The study area

**Fig. 1:**
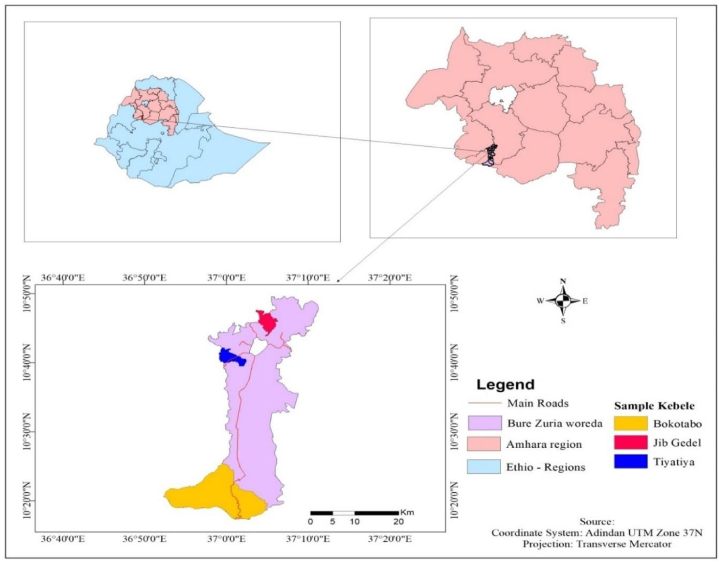
Map of the study area.

This research was investigated in the Bure Zuria district, northwestern Ethiopia. It lies between 10°18′N-10°49′29″N latitude and 36°52′1″E−37°7′9″E longitude (Fig. 1). It is 400 km far from the capital, Addis Ababa. Its altitude ranges from 700 to 2350 m.a.s.l. Three agro-ecologies are found in the district: midland (82%), lowland (10%), and highland (8%). The long-term maximum, minimum, and mean temperatures are 24, 14, and 19 °C, respectively. The rainfall pattern is bimodal and the annual maximum, minimum and mean rainfall are 1500, 1000, and 1250 mm, respectively. The population is about 104,784; with 13,940 and 1988 male and female-headed households, respectively. Households are mainly engaged in crop production (such as maize, finger millet, teff, wheat, barley, potato, pepper, onion rapeseed, been and field pea) and livestock rearing (cattle, sheep, goat, equine and chicken). However, the agriculture sector is suffering a lot from climate change and the frequency of crop failure in recent times. This makes the district the ideal place to study adaptation to climate change. The average cultivated land holding size of Bure Zuria district per household is estimated to be 1.6 ha [11]. This exceeds the national average cultivated land holding size of a household, 0.96 ha [23].

### 2.2 Data type, sources and collection methods

The primary data on the demographic, social, economic, institutional, and biophysical conditions of households were collected using household surveys, key informant interviews, focus group discussions, and field observation. Well-designed structured questionnaires were completed by respondents. Farmers were asked questions about their adaptation strategies and barriers for them to implement adaptation measures. The household questionnaire contains close-ended questions about the adaptation strategies practiced by farmers. However, farmers were allowed to specify the adaptation strategies they are practicing if it is different from the listed ones on the questionnaire. Twelve key informants: two persons from development agents, women, and elders across the three agro-ecologies participated in the interview. Key informant interviews were conducted to support the data, which were collected from the household survey about adaptation strategies and barriers. Focus group discussants comprising 8–10 persons from various socio-economic statuses were also involved. Focus group discussions were held to interact with a range of social groups and focus on group norms and dynamics around the issue being investigated. Besides, secondary data were acquired from published articles and the reports of the agriculture office and *kebele* (the smallest administrative division in Ethiopia) office.

### 2.3 Ethical statements and content validity evaluation

The authors follow all principles and ethical standards during this study. The Department of General Forestry (Specialization in Climate Change and Development) at Wondo Genet College of Forestry and Natural Resources, Hawassa University has approved this research. Before the data collection, households received the informed agreement, confidentiality, and privacy rights. Participants were informed about the objective of the study and their role during the data collection. Moreover, they were informed that the authors would be neutral during the process of data collection. The content validity process of the questionnaire was performed before the questionnaire has been distributed to the households. The expert review methodology was used to evaluate the validity of the questionnaire. Accordingly, two experts were involved to estimate the validity of a questionnaire systematically. This situation ensures that the questions are understandable to gather relevant information and address the objectives of the study.

### 2.4 Sampling design and sample size

A multi-stage sampling technique was used to select sample *kebeles* and households. Firstly, Bure Zuria district was chosen purposively because of the observed temperature increment and irregular rainfall pattern. Secondly, the district was stratified into three agro-ecologies like lowland, midland, and highland. Because the farmers’ adaptation measures differ from one agro-ecology to another agro-ecology. The livelihood strategies of farmers and the type of crop grown in different agro-ecologies are expected to vary. Thirdly, three *kebeles* (one from each agro-ecology) were selected randomly. Besides, a list of household heads and their total number in the selected *kebeles* were obtained from the *kebele* and district administration offices. Then, the sample size was determined by the formula indicated in Ref. [24] at 95% and 5% confidence level and precision level, respectively (Eq. (1)).

(Eq. 1) $$n=\frac{N}{1+N{\left(e\right)}^{2}}$$

where n refers to sample size, N refers to population size (total household heads size), and e stands for the precision level. Based on the above-mentioned formula, the required sample size was 190. Fourthly, the probability proportional to size technique was applied to decide the required sample at each agro-ecology. Finally, sample households were selected randomly using a simple random sampling technique. Accordingly, about 42, 59, and 89 households were involved in the lowland, midland and highland agro-ecologies (Table 1).

**Table 1:** Distribution of sample size in each agro-ecology.

Agro-ecologies	Household size	Sample size taken
Lowland	488	42
Midland	617	59
Highland	1019	89
**Total**	**2124**	**190**

### 2.5 Data analysis

#### 2.5.1 Descriptive analysis

Data were sorted, classified, coded and analyzed using descriptive statistics (frequency and percentage). Stata version 14 and EXCEL 2010 software were used for analysis.

#### 2.5.2 Econometric analysis

The multinomial logit (MNL) model was used to explore factors affecting farmers’ decision/use/choice of adaptation measures. Because it is extensively used in adoption decision studies involving multiple choices and is easier to compute. MNL is used when the response variables are more than two. To describe the MNL model, let y denote a random variable taking on the values {1, 2, …, J} for J, a positive integer, and let x denote a set of conditioning variables. Accordingly, y denotes adaptation strategies and x contains different household characteristics, economic variables, institutional and social factors, and P1, P2 … Pj as associated probabilities, such that P1 + P2 + … + Pj = 1 (Eq. (2)). This indicates how a certain change in *X* aﬀects the response probabilities (P(y = j/x), j = 1, 2 … J. Since the probabilities must sum to unity, P(y = j/x) is determined once the probabilities for j = 2 … J is known.

(Eq. 2) $$P\left(y=\frac{1}{x}\right)=1-\left(P2+P3+\ldots \mathrm{Pj}\right)$$

Let **x** be a 1 × K vector with first element unity. The MNL model has response probabilities:

(Eq. 3) $$p\;\left(y=j׀x\right)=\frac{\exp\left({x\beta }_{j}\right)}{\left[1+\sum_{h=1}^{j}\exp\left(x\beta_{h}\right)\;j=1,.\;..,j\right]}$$

Where $\beta_{j}$ is K × 1, j=1...J.

Unbiased and consistent parameter estimates of the MNL model in Eq. (3) require the assumption of Independence of Irrelevant Alternatives (IIA) to hold. In the MNL regression model, the parameter estimates provide simply the direction of the effect of the explanatory variables on the response variable, but estimates do not represent either the actual magnitude of change or probabilities. Differentiating Eq. (3) for the explanatory variables provides marginal effects of the explanatory variables given as (Eq. (4)):

(Eq. 4) $$\frac{\partial Pj}{\partial Xk}=P_{j}\;\left(\beta j_{k}-\sum_{j=1}^{j-1}p_{j}\beta_{jk}\right)$$

The marginal effects or marginal probabilities are functions of the probability itself and measure the expected change in the likelihood of a certain choice being made for a unit change in an independent variable from its average [25].

A multicollinearity test between explanatory variables was applied before estimating the model. This was done to meet the assumption of the Classical Normal Linear Regression Model (CNLM). Different methods are usually employed to detect the existence of multicollinearity problems. Accordingly, the variance inflation factor (VIF) (Eq. (5)) and contingency coefficient (CC) (Eq. (6)) were used to recognize the existence of multicollinearity for continuous and dummy variables, respectively.

(Eq. 5) $$VIF=\frac{1}{TOL}=\frac{1}{1-{Ri}^{2}}$$

where VIF is the variance inflation factor, TOL is tolerance, which is the inverse of VIF; *R*_*i*_^2^ is the coefficient of determination in the regression of one explanatory variable (*x*_*i*_) on another independent variable (*x*_*j*_). As per a rule of thumb**,** when the value of VIF is greater than 10, it is an indicator of the existence of a multicollinearity problem [26].

(Eq. 6) $$CC=\frac{\sqrt{X^{4}}}{N+X^{4}}$$

where CC = contingency coefficient, *X*^2^ = chi-square, N = total sample size. If the contingency coefficient test value exceeds 0.8 for those dummy variables, it indicates a multicollinearity problem [26]. Thus, all variables except farm size were considered in the analysis.

### 2.6 Definition of variables and hypothesis

The response variables are climate change adaptations that farmers implement in the study area. As stated by Ref. [27], many African countries use adjusting planting times, changing crop varieties, irrigation, and soil conservation practices as climate change adaptation mechanism. Moreover, the description of explanatory variables is presented below in Table 2.

**Table 2:** Description of explanatory variables.

Explanatory variables	Variable type	Variable measurement	Expected effect(sign)
Agro-ecology	Categorical	0 if midland, 1 if lowland and 2 if highland	+/−
Age	Continuous	Year	+
Sex (Male)	Dummy	0 if male, 1 otherwise	+
Level of education	Continuous	Year	+
Family size	Continuous	Number	+/−
Farm income	Continuous	Birr	+
Off-farm income	Continuous	Birr	+/−
Number of livestock	Continuous	Tropical Livestock Unit (TLU)	+
Credit access	Dummy	1 if there is access, 0 otherwise	+
Market access	Dummy	1 if there is access, 0 otherwise	–
Access to climate information	Dummy	1 if there is access, 0 otherwise	+
Frequency of Extension contact	Continuous	Number	+

## 3 Results and discussion

### 3.1 Farmers’ climate change adaptation measures in different agro-ecologies

**Fig. 2:**
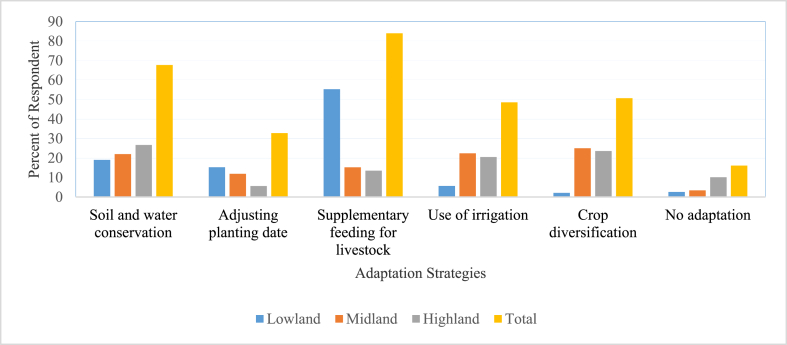
Distribution of farmers' adaptation measures by agro-ecologies.

Different adaptation measures were implemented to alleviate the current climate change-related hazards. Crop diversification, soil and water conservation practices, adjusting planting dates, irrigation, and supplementary feeding for livestock were used as adaptation measures to combat climate change. Farmers living in diverse agro-ecologies are likely to implement different adaptation options against the impacts of climate change [22]. Fig. 2 depicts that soil and water conservation practices (26.7%) were the most widely used adaptation strategies followed by crop diversification (23.6%). In contrast, adjusting planting dates (5.6%) was the least-practiced adaptation response by farmers in highland agro-ecology of the district. This could probably be associated with the fact that highland areas are likely more vulnerable to flooding hazards and soil erosion from excessive rainfall. This result is in line with the finding of [28] who reported that terracing was the most widely used adaptation strategy in the highland areas of Ambassel district in Ethiopia. Moreover [28,29], reported that adjusting planting dates and terracing were the most practiced adaptation measures by farmers in lowland and highland areas, respectively. In midland areas, crop diversification (25%) was the main adaptation response. The results also indicated that supplementary feeding for livestock (56%) was the most implemented adaptation response followed by soil and water conservation practices (19%), whereas crop diversification (2.1%) was the least common adaptation response to the adverse impacts of climate extremes by farmers in lowland agro-ecology. This result contradicts with the finding of [30] who reported that the possibility of using crop diversification was very high in lowland areas. Key informants and focus group discussants also reported that soil and water conservation practices and feeding for livestock were the most widely used adaptation measures in highland and lowland areas of the district.

Farmer's decision-making process to choose a certain climate change adaptation measure involves trade-offs and synergies. According to Ref. [31], trade-offs encompass the balancing of factors that cannot be achieved at the same time or in combination. Soil and water conservation practices are essential to reduce run-off and erosion, thereby boosting crop growth. In contrast, it involves trade-offs of high construction and installation cost. Supplementary feeding for livestock plays a paramount role in securing the nutritional demand of vulnerable people. The majority of focus group discussants argued that irrigation has a positive effect in terms of increasing water for crop and livestock production. Irrigation indeed increases access to water for growing diverse crops, thereby maximizing crop production [32]. Nevertheless, irrigation demands huge initial investment and operational costs. It also has maladaptive outputs of water scarcity for low-lying farmers. Besides, some irrigation systems are not environmentally friendly. Crop diversification is practiced by farmers to increase yields and income. Farmers also practiced intercropping with legumes. This helps to improve soil fertility [33]. However, it increases the financial burden on farmers. Besides, farmers combine different crop varieties with agricultural chemicals, which results in a threat to the environment and human beings. Hence, it is vital to address potential trade-offs before the implementation of each climate change adaptation measure.

Women are an integral part of labor-demanding activities in farming. However, their labor contributions are often underestimated. Adaptation strategies to climate change were largely influenced by gender in the study area. Soil and water conservation and supplementary feeding for livestock were adopted by more men than women. Thus, it requires addressing the trade-offs which are caused by gender inequalities. Key informants witnessed that irrigation and crop diversification were also mainly the role of men. They also reported that the unequal distribution of rights and lack of sufficient educational opportunities contributed to gender inequality. According to Ref. [34], a lack of access to information and education can limit women's adaptive capacity to climate change impacts. Besides, focus group discussants stated that women's participation in the decision-making process is very low. This situation exacerbates their vulnerability to climate change hazards. Vulnerability to climate change can be aggravated by gender inequality [35]. Hence, it is vital to strengthen gender considerations in adaptation planning.

### 3.2 Barriers faced by farmers to implementing adaptation measures in different agro-ecologies

**Fig. 3:**
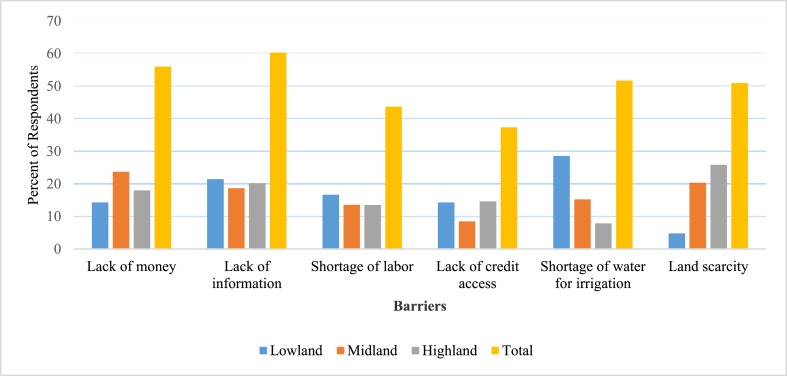
Barriers faced by farmers to implementing adaptations in different agro-ecologies.

The major constraints that made farmers’ life very difficult to withstand climate change-induced hazards were lack of money, lack of information, shortage of labor, lack of credit access, shortage of water for irrigation, and land scarcity (Fig. 3). The result of this study agrees with the findings of [21,28]in Ethiopia. Farmers identified land scarcity (25.84%) and shortage of water for irrigation (28.57%) as the major barriers to implementing adaptation measures in highland and lowland agro-ecologies, respectively. This might be attributed to the large population size and density in highland areas. Furthermore, the low forest coverage and climate-related extremes could be the reasons for the shortage of water for irrigation in lowland agro-ecology. Key informants and group discussants also witnessed that the below-mentioned barriers to implementing adaptation measures were common.

### 3.3 Determinants of farmers' adaptation strategies to climate change

Table 3 depicts the key factors that influence farmers’ choice of adaptation measures. The likelihood ratio statistics (LR chi-square (60) = 356.33) are highly significant P < 0.0000). The main limitation of this study was the incorporation of only twelve explanatory variables in the multinomial logit regression model because of time constraints.

**Table 3:** Parameter estimates of the MNL model.

Variable	Crop Diversification		Soil and water conservation practice		Adjusting planting date		Use of irrigation		Supplementary feeding for livestock	
	Coefficients	Std. error	Coefficients	Std. error	Coefficients	Std. error	Coefficients	Std. error	Coefficients	Std. error
Agro-ecology (highland)	−1.717	1.185	2.497*	1.290	−0.130	2.450	0.513	1.269	2.862**	1.325
Agro-ecology (lowland)	2.266	1.753	3.810**	1.787	9.792***	2.323	1.311	0.990	8.243***	2.011
Sex	0.438	0.804	−0.089	1.022	1.509	3.888	0.445	0.896	−0.307	0.946
Education level	0.807***	0.284	1.042***	0.274	2.107***	0.663	0.848***	0.298	1.074***	0.292
Age	0.307***	0.068	−0.013	0.069	3.293***	1.162	0.212***	0.077	0.276***	0.074
Active labor	−0.078	0.338	−0.632	0.435	−1.762**	0.893	−0.232	0.364	−0.707*	0.398
Livestock number (TLU)	−0.317*	0.171	−0.183	0.196	0.803	0.666	−0.172	0.162	−0.558***	0.209
Farm income	0.206	0.543	0.402	0.544	−3.724	2.571	1.333	0.850	1.189	0.738
Off farm income	0.074	0.110	−0.277**	0.114	−1.525**	0.665	−0.121	0.133	0.0002	0.121
Frequency of extension.contact	0.065	0.075	0.091	0.070	1.412**	0.603	0.032	0.084	−0.011	0.084
Access to clim.info.	−0.404	0.672	−1.018	0.796	1.388	1.230	−0.470	0.781	−0.422	0.797
Access to credit (No)	−0.662	0.692	−1.094	0.789	−0.475	2.047	−0.360	0.774	−0.502	0.794
Distance to the market	0.396	0.253	−0.262	0.277	1.359*	0.748	0.187	0.277	−0.759	0.318
Cons.	16.615***	5.552	−0.778	5.297	−172.798**	70.108	−22.963***	7.851	−15.406**	6.967
Base category	No adaptation									
Number of observations	190									
LR chi2(60)	356.33									
Log likelihood	−157.56599									
Prob >	chi2 0.0000									
Pseudo R2	0.5307									

***, **,* Significant at 1%, 5%, and 10% probability level, respectively.

#### 3.3.1 Agro-ecology

The MNL model results showed that farming in lowlands significantly improves the chance of adjusting planting dates and supplementary feeding for livestock by 34.1 and 30.6% at a 5% significance level, respectively (Table 4). This result is also confirmed by the assessment result of this study (Fig. 2) which showed that more than half of farmers (56%) used supplementary feeding for livestock to adapt to climate change impacts. However, farming in the lowlands significantly reduces the chance of crop diversification and irrigation by 38 and 8.9%, respectively, as compared with farming in the midland. Farmers in diverse agro-ecologies employ different adaptation methods [22]. Moreover, farming in highlands significantly improves the chance of implementing soil and water conservation and supplementary feeding for livestock by 15.3 and 16.5%, respectively. Nevertheless, farming in highlands declines the likelihood of crop diversification by 34.7%. Living in highland agro-ecology increases the chance of employing adaptation strategies [36]. The difference might be due to the diﬀerence in soil, climate, and other natural resources and experiences of climate-related stress. The finding [37] also stated that farmers in different agro-ecologies have different choices of adaptation.

**Table 4:** Marginal effects from the MNL model.

Explanatory variables	Crop diversification		Soil and water conservation practices		Adjusting planting dates		Use of irrigation		Supplementary feeding for livestock	
	Coefficients	Std. error	Coefficients	Std. error	Coefficients	Std. error	Coefficients	Std. error	Coefficients	Std. error
Agro-ecology (highland)	−0.347***	0.086	0.153**	0.061	0.0005	0.026	0.065	0.061	0.165***	0.046
Agro-ecology (lowland)	−0.380***	0.094	−0.020	0.058	0.341***	0.032	−0.089*	0.053	0.306***	0.056
Sex	0.033	0.071	−0.014	0.065	0.019	0.058	0.022	0.053	−0.048	0.059
Education level	−0.004	0.014	0.029***	0.007	0.018**	0 .008	0.004	0.010	0.019*	0.010
Age	−0.008	0.008	−0.017***	0.003	0.046***	0.012	−0.005	0.003	−0.001	0.004
Active Labor	0.045**	0.023	−0.026	0.023	−0.023**	0.011	.006	0.017	−0.031	0.020
Livestock number TLU)	−0.018	0.016	0.005	0.012	0.017**	0.008	0.006	0.010	−0.032**	0.013
Farm income	−0.032	0.054	−0.012	0.032	0.067**	0.033	0.082	0.057	0.074	0.048
Off-farm income	0.030***	0.010	−0.019***	0.006	−0.023***	0.007	−0.005	0.008	0.011	0.007
Frequency of extension contact	−0.007	0.007	0.004	0.003	0.021***	0.007	−0.003	0.005	−0.010*	0.005
Access to climate info.	0.079	0.069	−0.045	0.076	0.043	0.047	−0.034	0.053	−0.027	0.054
Access to credit (No)	−0.027	0.058	−0.054	0.046	0.001	0.029	0.016	0.046	0.169**	0.068
Market access	0.054***	0.016	−0.012	0.015	0.020**	0.009	0.012	0.013	−0.075***	0.017

***, **,* Significant at 1%, 5%, and 10% probability level, respectively.

#### 3.3.2 Education level

A one-year increment in the level of education increases the chance of employing soil and water conservation, adjusting planting dates and supplementary feeding for livestock by 2.9, 1.8, and 1.9% (at 1%, 5% and 10% significance level), respectively (Table 4). The reason might be literate farmers are anticipated to adopt new technologies based on their awareness of the potential benefits of adaptation measures [12,38].

#### 3.3.3 Age

The result indicated that age had a positive and significant correlation with adjusting planting dates. A one-year increase in age raises the chance of adjusting planting dates by 4.6% (Table 4). The probable reason could be age may likely give farmers the experience that enables them to make a better assessment of the risks involved in the investment of adaptation options. The finding is supported by Ref. [28] who noted that households who have long experience in farming can predict the trends of crop production better than young households. In contrast, age shows a negative correlation with soil and water conservation practice. One-year increase in age lessens the possibility of soil and water conservation by 1.7 times greater relative to households that do not take any adaptation measures. This infers that older farmers have a lower chance to implement soil and water conservation adaptation measures than younger farmers. Thus, younger households are more energetic than older households in employing soil and water conservation practices. Because soil and water conservation practices require more labor. The result agrees with [22] who noted that younger households are more expected to practice soil and water conservation adaptation measures than older ones.

#### 3.3.4 Active labor

Table 4 depicts that active labor was negatively correlated with households’ decision to adapt to adjusting planting dates. A person increase in active labor would cause a reduction in the chance of adjusting planting dates by 2.3%. This might be because farmers who had large active labor might be enforced to divert the force partially to non-farm activities to make income and reduce the consumption pressure. The result agrees with the findings of [1,39,40] who stated that family size had a significant and adverse effect. However, active labor is positively related to crop diversification.

An increase in the number of economically active household sizes by one person raises the chance of implementing crop diversification by 4.5%. This might be associated with higher labor endowment, which would be helpful to accomplish different agricultural activities like diversifying farm products. This finding is supported by Ref. [16] who stated that family size both positively and negatively influences farmers’ adaptation strategies.

#### 3.3.5 The number of livestock (TLU)

TLU had a positive and significant impact on adjusting planting dates. A unit increase of TLU would raise the likelihood of using adjusting planting dates by 1.7% (Table 4). The reason could be related that livestock has a vital role starting from land preparation to planting crops. The finding of [41] revealed that livestock production has a positive association with adjusting planting season, integrating crops with livestock rearing, and soil and water conservation measures. However, livestock ownership showed a significant and adverse relationship with supplementary feeding for livestock. Moreover, a unit rise in TLU would reduce the possibility of using supplementary feeding for livestock by 23.2%. The reason could be households with huge TLU cannot provide supplementary forage for their livestock due to space and time limitations. Similarly [16], revealed that TLU negatively affects adaptation measures to climate change at a 1% significance level.

#### 3.3.6 Farm income

The model result indicated that on-farm income had a positive and significant impact on adjusting planting dates. The marginal eﬀect depicted that one-birr increase in farm income raises the likelihood of adopting the aforementioned adaptation measure by 6.7%. The reason could be farmers with high farm income might have a higher chance to invest more time in agricultural tasks to reduce the influences of climate change and variability. This finding is supported by Ref. [21] who stated that income had significant positive influences on households' adaptation strategies. However, it contradicts the finding of [36] who revealed that income had significant negative impacts on households' adaptation strategies.

#### 3.3.7 Off-farm income

It increased the chance of implementing crop diversification significantly. A unit increase in non-farm income raises the chance of implementing crop diversification by 3%. The reason might be farmers with higher off-farm income might have the additional financial power to diversify crops and enhance their farm productivity. According to Ref. [38], off-farm income improves farmers' financial position and enables them to purchase farm inputs. In contrast, off-farm income showed a negative correlation with adjusting planting dates and soil and water conservation. When off-farm income increased by a unit, it decreased the chance of implementing soil and water conservation measures and adjusting planting dates by 1.9 and 2.3%, respectively. The reason could be farmers with high off-farm income might have a lower possibility to invest more time in agricultural tasks to reduce the influence of climate change risks. Similarly [42], stated that as the farmers' income from off-farm activities increased they devote less time to farming activities hence it could negatively affect the households’ adaptation to climate change.

#### 3.3.8 Frequency of extension contact

The frequency of extension visits had positive and significant impacts on adjusting the planting dates as an adaptation option at a 1% significance level. Table 4 depicts that a unit increase of extension contact raises the chance of farmers using adjusting planting dates by 2.1%, unlike households without access to extension services. Similarly [41], stated that an increase in extension contact raises the chance of employing soil and water conservation, tree planting, crop diversification, and adjusting planting dates. It is usually reported as a crucial factor affecting farmers’ climate adaptation measures [12,20,36]. In contrast, extension visits had a significant and adverse impact on the chance of using fertilizer as an adaptation measure. Besides, a unit increase of it reduces the chance of providing supplementary feeding for livestock by 1%. The reason could be extension contacts could not be necessarily on livestock production but rather on crop production. This indicates that extension services on livestock production should be promoted jointly with other extension services.

#### 3.3.9 Access to credit

Access to affordable credit increases the financial resources of farmers and their capability to gain transaction costs related to different adaptation measures [43]. It influences supplementary feeding for livestock positively and significantly at a 5% significance level. The households that had better credit access were more expected to employ supplementary feeding for livestock by 16.9%. Supplementary forage for livestock requires capital investment, which most ordinary households could not afford. Therefore, leveraging the cash shortage of households through credit might encourage farmers to provide supplementary feeding for livestock. However, the result contradicts [40] who revealed that credit access adversely affects farmers’ adaptation strategies.

#### 3.3.10 Market access

It had a positive and significant impact on adopting crop diversification and adjusting planting dates. Being a resident near the market center increased the chance of implementing crop diversification and soil and water conservation adaptation measures by 5.4 and 2%, respectively. Thus [41], noted that easy access to the market rises the chance of implementing crop diversification.

## 4 Conclusions and policy implications

### 4.1 Conclusions

This study explored the factors affecting farmers' adaptation mechanisms using a multinomial logit regression model in the Burie Zuria district, Ethiopia. The findings of this study showed that diverse adaptation measures were mainly implemented by farmers to combat climate change impacts in different agro-ecologies. These comprise crop diversification, adjusting planting dates, soil and water conservation measures, supplementary feeding for livestock, and using irrigation. Soil and water conservation practices are the most widely used adaptation strategies in highland areas, unlike lowland areas. Because the steepness of farmlands and erodible soils increase farmers' vulnerability to flood hazards in highland areas than in lowland areas. Nevertheless, supplementary feeding for livestock was found the main adaptation strategy in lowland areas than highland areas. This is because the livelihood of farmers in the lowland areas is more dependent on livestock, unlike farmers in highland areas. Land scarcity and shortage of water for irrigation were the principal barriers to implementing adaptation measures in highland and lowland areas of the district, respectively. Furthermore, farmers' decision to choose effective adaptation measures was affected by different socioeconomic and demographic characteristics in the district. Multinomial logit regression results revealed that farmers’ choice of adaptation measures was influenced by agro-ecologies, education level, age, active labor, TLU, farm income, off-farm income, frequency of extension contact, access to credit, and market access at a 5% significance level in the study area.

### 4.2 Policy implications

The agriculture office of the district shall provide advanced technical support on the integrated soil and water conservation practices for farmers living in the highland agro-ecology. This situation would help them to better adapt to climate change hazards. The government should work in consortium with non-government organizations to maximize the quality and quantity of supplementary feeding supply for livestock and reduce the adverse impacts of climate change in lowland agro-ecology. Moreover, since land scarcity is found the main barrier to implementing adaptation measures in the highland area, farmers should focus on the adaptation measures, which can be implemented on limited land like agroforestry practices. The policymakers in the agriculture sector shall give due attention to the interventions of improving the accessibility of adult education, farmers' livestock holding, farm income, off-farm income, affordable credit and market access when developing adaptation policies. Development Agents shall increase contacts with farmers to provide updated information regarding climate change and support adaptation efforts that have been made. It is also essential to offer support and affordable credit to farmers for adaptation investments in the preparation of supplementary feeding for livestock. This would be very essential to enhance farmers’ adaptive capacity against climate-related extremes. It is suggested that investigations on the climate change-induced gender-differentiated impacts shall be conducted to design all-inclusive and effective responses.

## Author contribution statement

Esubalew Molla, Yoseph Melka and Getnet Alemu: Analyzed and interpreted the data; Wrote the paper.

## Data availability statement

The data that has been used is confidential.

## Additional information

No additional information is available for this paper.

## Declaration of competing interest

The authors declare that they have no known competing financial interests or personal relationships that could have appeared to influence the work reported in this paper.
